# Hydrogen Embrittlement Resistance of an Austempered Martensite/Bainite Dual-Phase Steel

**DOI:** 10.3390/ma19173616

**Published:** 2026-08-25

**Authors:** Fengping Zhao, Zhilin Guo, Zhaojing Zeng, Xiaofei Guo

**Affiliations:** 1 School of Materials Science and Engineering, Shanghai University, Shanghai 200444, China; 2 State Key Laboratory of Advanced Special Steel, Shanghai University, Shanghai 200444, China

**Keywords:** ultra-high-strength fastener steel, austempering below *M_s_* temperature, martensitic/bainitic dual-phase, carbide precipitation, hydrogen embrittlement

## Abstract

Hydrogen embrittlement (HE) behavior of 31CrMoNiNbV steel subjected to austempering below the *M_s_* temperature was investigated. The material exhibited a martensitic/bainitic dual-phase microstructure, with uniformly dispersed V- and Nb-rich MC carbides as well as Cr-rich M_3_C carbides precipitated in the matrix. It achieved an ultimate tensile strength of 1688 MPa and an elongation to fracture of 10.7%. Slow strain rate tensile tests revealed that the HE susceptibility index reached 13.5%. Thermal desorption spectroscopy and hydrogen permeation tests showed that the martensite/bainite phase boundaries and the dispersed MC/M_3_C carbides acted as effective hydrogen-trapping sites, lowering the hydrogen diffusivity and alleviating local hydrogen accumulation in stress-concentrated regions. Microstructural analysis further indicated that the dual-phase structure may simultaneously mitigate the hydrogen-enhanced localized plasticity and hydrogen-enhanced decohesion mechanisms, through combined stress relaxation at martensite/bainite interfaces and hydrogen immobilization by carbide traps.

## 1. Introduction

Driven by the growing demand for lightweight design without compromising structural integrity in automotive and energy sectors, ultra-high-strength fastener materials with long-term service stability are increasingly required [1,2,3]. However, hydrogen embrittlement (HE) becomes a critical bottleneck limiting the safe service of ultra-high-strength steels, as local enrichment of diffusible hydrogen at microstructural heterogeneities can trigger catastrophic brittle fracture under stresses far below the nominal tensile strength [4,5]. To mitigate this risk, extensive efforts have been devoted to microstructural design, chemical composition optimization, and process control throughout the mechanical processing chain [6,7,8,9].

Currently, most high-strength fastener steels rely on a tempered martensite matrix, which often exhibits insufficient HE resistance under corrosive service environments [10,11]. In tempered martensite, continuous or semi-continuous cementite films along prior-austenite and lath boundaries act as weak, hydrogen-prone interfaces, and hydrogen segregation at these sites triggers grain boundary decohesion and drives preferential crack propagation along prior-austenite grain boundaries. In contrast, bainite (particularly granular and lower bainite) contains carbides dispersed as fine and discontinuous particles within the ferrite matrix rather than as continuous brittle films along boundaries. Moreover, the abundant ferrite/cementite interfaces, interlath retained-austenite films, and relative high dislocation density act as reversible hydrogen-trapping sites, which can retard hydrogen transport and reduce the local hydrogen concentration at potential crack initiation sites and grain boundaries [12,13]. The bainitic/martensitic dual-phase microstructure can further provide a synergistic balance between strength and toughness, making it well suited to fastener applications that require both high stiffness and adequate toughness [14,15]. Ju et al. [16] demonstrated that in lower bainite/martensite (LB/M) duplex steels for construction machinery, the fine, thermally stable carbides within lower bainite suppress lath coarsening and matrix softening during prolonged tempering, such that after 100 h at 600 °C, the LB/M duplex structure (44% lower bainite) retained higher hardness than fully martensitic steel. Chakraborty et al. [17] further showed that in SAE52100 bearing steel, an optimized LB/M duplex microstructure obtained by austempering at 270 °C achieved a tensile strength of 2250 MPa together with an impact energy of 53 J, attributing this synergy to plastic constraint between the soft bainite and hard martensite phases.

Beyond their beneficial effects on mechanical properties, LB/M duplex microstructures have also been shown to improve HE resistance. Zhang et al. reported that, in as-hot-rolled bainitic rail steel, coarse blocky M/A constituents promoted hydrogen accumulation and microcrack initiation, whereas tempering at 500 °C decomposed these constituents and markedly improved HE resistance with only a minor loss in strength [18]. Qi et al. [19] compared 1400 MPa-grade 42CrMoVNb fastener steel with tempered-martensite and austempered LB/M microstructures at comparable strength levels, and found that the HE susceptibility index of the LB/M steel (20.7%) was substantially lower than that of tempered martensite steel (35.1%). This improvement was attributed to the absence of coarse grain boundary carbides, reduced dislocation density, and the presence of dense ferrite/cementite interfaces within bainite, which act as reversible hydrogen-trapping sites, retard hydrogen transport and suppress crack initiation. These findings indicate that introducing lower bainite into a tempered martensite matrix is a promising strategy for achieving favorable balance between strength and toughness while simultaneously improving HE resistance [20,21].

This work investigated HE behavior in 31CrMoNiNbV steel subjected to austempering below the *M_s_* temperature, which achieved martensite/bainite microstructures and tensile strength above 1600 MPa through substantial carbide precipitation. By performing slow strain rate tensile (SSRT) testing, fracture surface analysis, and microstructural characterization near the fracture head region, the underlying HE mechanisms were elucidated, providing experimental and theoretical guidance for microstructure and heat-treatment optimization of ultra-high-strength fastener steels with improved HE resistance.

## 2. Materials and Methods

### 2.1. Materials and Heat-Treatment Procedures

The investigated material was a medium-carbon steel (designated as 31CrMoNiNbV), with Cr, Ni, and Mo as the principal alloying elements, microalloyed with Nb and V to promote grain refinement and precipitation hardening. The chemical composition is listed in Table 1. The martensite start temperature (*M_s_*) of the steel is 354 °C. Heat treatment was carried out in two stages: (i) normalizing at 1060 °C for 40 min in a Nabertherm LT 24/12 muffle furnace followed by air cooling; (ii) austenitizing at 1040 °C for 60 min, immediate transfer to a salt bath furnace held at 270 °C for 8 h (austempering below *M_s_* temperature), and final oil quenching to room temperature.

### 2.2. Microstructural Characterization

Microstructure was characterized by scanning electron microscopy (SEM, ZEISS Gemini460, Oberkochen, Germany) after etching in a 4 vol.% nitric acid–ethanol solution for 5 s. Crystallographic features were characterized by electron backscatter diffraction (EBSD, Oxford Instruments, Abingdon, UK) with a step size of 0.1 µm. Correlative EBSD and electron channeling contrast imaging (ECCI) were combined to reveal crack propagation paths and to assess the influence of grain orientation and grain/phase boundaries on crack behavior in the fracture-tip region. Lath morphology and precipitated carbides were characterized by transmission electron microscopy (TEM, Talos F200X, Thermo Fisher Scientific, Hillsboro, OR, USA) equipped with an energy-dispersive spectrometer (XFlash Detector 5030, Bruker AXS, Karlsruhe, Germany). TEM foils were prepared by twin-jet electropolishing (Tenupol-5, Struers ApS, Ballerup, Denmark) in a 10% perchloric acid–ethanol electrolyte at −30 °C, followed by final polishing with a precision ion polishing system (Gatan 691 PIPS, Getan, Inc, Pleasanton, CA, USA). X-ray diffraction (XRD, Smartlab, Rigaku Corportation, Akishima, Japan) measurements were performed using Cu-Kα radiation at 40 kV over a 2θ range of 20° to 110° at a scanning speed of 1°/min, focusing on the (110)α, (211)α, and (220)α diffraction peaks.

### 2.3. Mechanical Properties

Tensile and Charpy V-notch (CVN) impact specimens were machined along the rolling direction. Tensile tests were performed with a 300 kN universal testing machine (Instron 5985, Instron, Norwood, MA, USA) at a strain rate of 5 × 10^−4^ s^−1^, using smooth cylindrical specimens with a gauge length of 35 mm and a diameter of 5 mm. Low-temperature impact tests were conducted at −40 °C using an impact testing machine (Instron Mpx750, Instron, Norwood, MA, USA) with V-notched specimens (10 mm × 10 mm × 55 mm, notch depth of 2 mm). Hardness was measured on a Rockwell hardness tester (Wilson RH2150, Buehler, Lake Bluff, IL, USA) with a diamond indenter under a load of 150 kgf and a dwell time of 2 s. All mechanical tests were repeated at least three times.

### 2.4. Slow Strain Rate Testing

**Figure 1:**
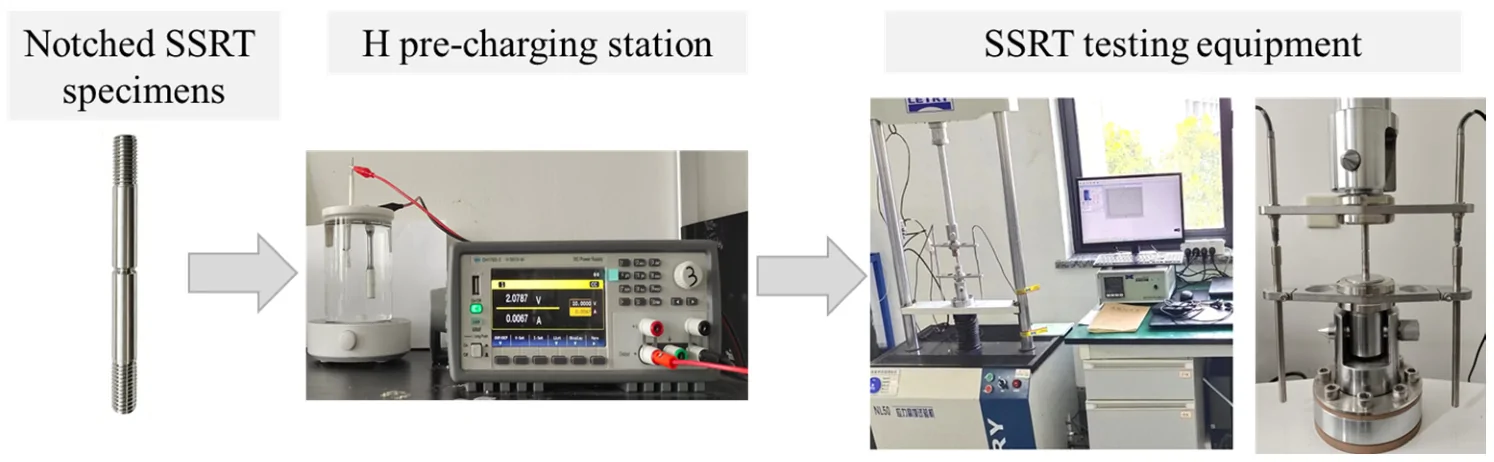
The set-up of hydrogen pre-charging and SSRT testing.

SSRT tests were conducted using a stress corrosion testing machine (LETRY WDML-50, Xi’an, China) at a crosshead speed of 0.005 mm/min. V-notched tensile specimens with a notch root radius of 0.15 ± 0.02 mm were used, corresponding to a stress concentration factor K_t_ of 3.5 [22]. The effects of precharged hydrogen on tensile behavior were evaluated. For hydrogen recharging, specimens were cathodically charged in 0.1 mol/L NaOH aqueous solution at a current density of 1 mA/cm^2^ for 24 h. To minimize hydrogen loss during testing, the charged specimens were immediately electroplated with a nickel layer at 10 mA/cm^2^ for 10 min in a solution containing 300 g/L NiSO_4_·6H_2_O, 82 g/L NiCl_2_·6H_2_O, 30 g/L H_3_BO_3_, and 0.5 g/L C_12_H_25_SO_4_Na. The set-up of hydrogen pre-charging and SSRT testing facilities is shown in Figure 1. All SSRT tests were repeated three times to ensure reproducibility. HE susceptibility was quantified using the hydrogen embrittlement index (HEI), defined based on the relative loss of notch tensile strength [2]:

(1) $$HEI\left(\%\right)=\frac{\sigma_{bN0}-\sigma_{bN}}{\sigma_{bN0}}\times 100\%$$

where $\sigma_{bN0}$ and $\sigma_{bN}$ are the notched tensile strength of the uncharged and hydrogen-charged specimens respectively. Fracture surfaces were examined by the SEM described in Section 2.2. The crack propagation path in the cross-sectional region beneath the notch was characterized by correlative EBSD and electron channeling contrast imaging (ECCI) analysis.

### 2.5. Hydrogen Content Measurement

Hydrogen content was measured on specimens subjected to the same pre-charging conditions as the SSRT specimens (Section 2.4). Diffusible hydrogen content was determined by a thermal desorption spectrometer (TDS, G8 Galileo, Bruker AXS, Karlsruhe, Germany) fitted with a quadrupole mass spectrometer, using heating rates of 10, 20, and 30 °C/min. The apparent activation energy (*E*a) for hydrogen detrapping was calculated from the shift in desorption peak temperature with heating rate according to the Kissinger equation [23]:

(2) $$E_{a}=-R\frac{\partial \left(\ln\frac{\Phi }{T_{p}^{2}}\right)}{\partial \left(\frac{1}{T_{p}}\right)}$$

where Φ is the heating rate, T_p_ is the peak desorption temperature and R = 8.314 J K ^−1^ mol^−1^ is the gas constant. Peak-fitting was performed using Gaussian deconvolution. Each TDS measurement was repeated twice to ensure the reliability and repeatability of the experimental results.

### 2.6. Hydrogen Permeation Test

Hydrogen diffusion behavior was characterized using a Devanathan–Stachurski double electrolytic cell controlled by a dual-channel electrochemical workstation (CS2350M, Wuhan Corrtest Instruments Corp., Wuhan, China) [24]. Disk-shaped specimens (Φ30 mm × 1 mm) were mechanically polished to a 0.6 µm surface finish. The oxidation side was electrochemically nickel-plated to stabilize the background current at the applied anodic potential. The anodic compartment, containing 0.2 mol/L NaOH, was polarized at 0.2 V (vs. Hg/HgO) until the residual current dropped below 1 μA, while the cathodic (charging) compartment was subjected to galvanostatic hydrogen charging under the same conditions used for pre-charging (Section 2.4). Both electrolytes were continuously purged with high-purity N_2_ before and during testing to minimize interference from dissolved oxygen.

A cyclic permeation method was used to distinguish reversible and irreversible hydrogen trap densities [22,25]. In the first charging cycle, hydrogen charging was maintained until the anodic current reached a steady-state value. The specimen was then held at room temperature for 24 h to allow diffusible hydrogen to desorb, after which a second charging cycle was performed under identical conditions. The anodic current (*I*_a_) was recorded at 1 s intervals to construct the hydrogen permeation curves. The apparent hydrogen diffusion coefficient *D_app_* (cm^2^·s^−1^) was calculated from the permeation transients using the time-lag method [26]:

(3) $$D_{\mathrm{app}}\;=L^{2}/6t_{0.63}$$

where *L* is the specimen thickness (cm), and t_0.63_ (s) is the time required for the oxidation current density to reach 63% of its steady-state value *I*_ss_ (A). The apparent hydrogen solubility *C_0app_* (mol·cm^−3^) beneath the membrane surface at the hydrogen entry side can be calculated using

(4) $$C_{0app}=J_{ss}L/D_{app}$$

where $J_{\mathrm{ss}}$ (mol·cm^−2^·s^−1^) is the steady-state hydrogen permeation flux, which can be calculated by the following equation [27]:

(5) $$J_{\mathrm{ss}}=I_{\mathrm{ss}}/\mathrm{AF}$$

where A is the exposed area of the sample (1.76625 cm^2^), and F is the Faraday constant (96,485 C·mol^−1^). The density of hydrogen trap sites (*N*_T_) can be expressed as [28]

(6) $$N_{T}=N_{L}\times (\frac{D_{L}}{D_{\mathrm{app}}}-1)\times e^{-(\frac{E_{b}}{RT})}$$

where *N*_L_ is the density of the interstitial sites, 5.1 × 10^23^ sites·cm^−3^ [28]; D_L_ is the value of the lattice diffusion coefficient, 1.45 × 10^−5^ cm^2^·s^−1^ in martensitic steel [29]; E_b_ is the hydrogen trap binding energy, *E*_b_ = 27 kJ·mol^−1^; and R is the gas constant, 8.314 J·mol^−1^·K^−1^. The density of irreversible hydrogen traps (*N*_ir_) can be obtained from the difference between the hydrogen trap densities calculated from the two cyclic hydrogen permeation transients.

## 3. Results

### 3.1. Microstructure Characterization and Mechanical Properties

**Figure 2:**
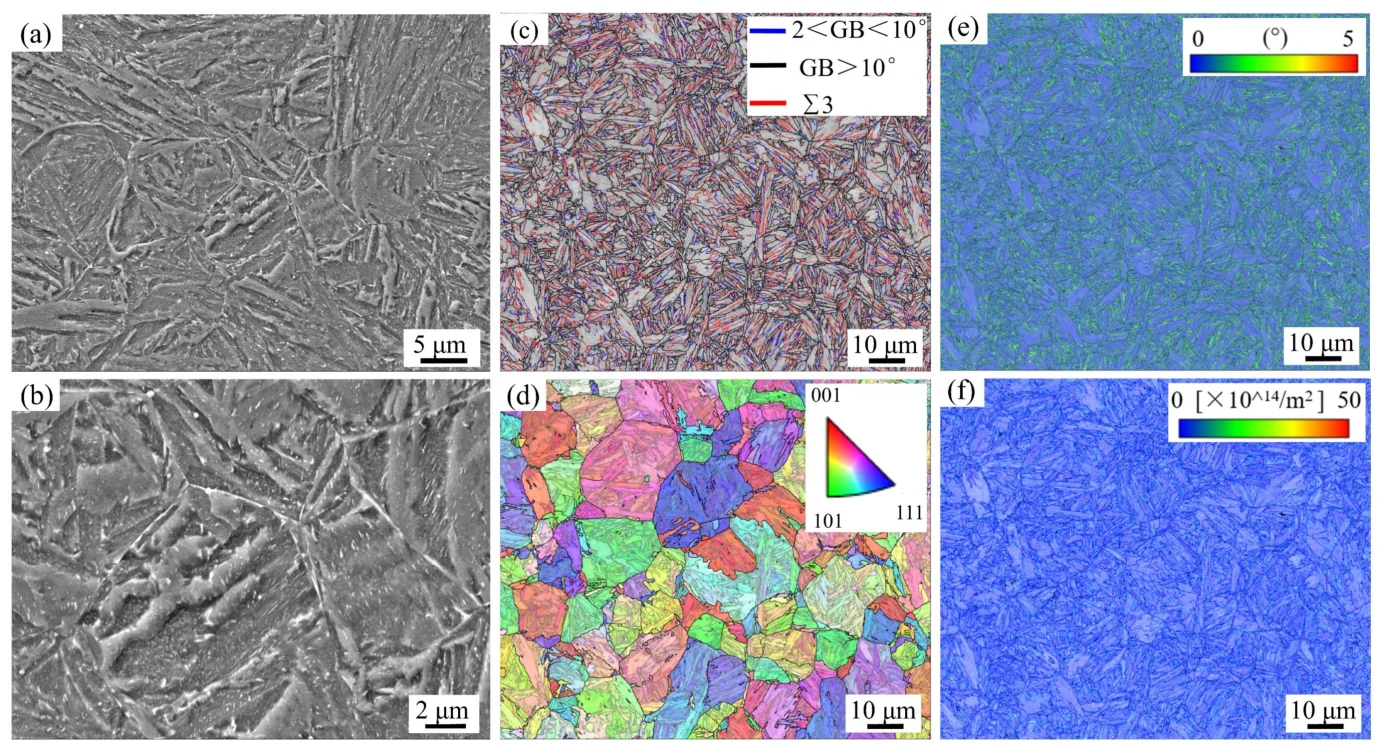
Microstructural characterization of the austempered experimental steel: (**a**) low-magnification SEM morphology; (**b**) high-magnification SEM morphology. EBSD analysis results: (**c**) grain boundary distribution map; (**d**) inverse pole figure (IPF) map; (**e**) KAM map; (**f**) GND density distribution map.

The microstructure of the austempered steel is shown in Figure 2. The specimen consists predominantly of a martensitic and bainitic dual-phase microstructure, as shown in Figure 2a,b. A large number of fine carbides are dispersed throughout the microstructure, exhibiting a small size and uniform distribution. Figure 2c–f present the EBSD results, illustrating the grain boundary character and crystallographic orientation distribution. In Figure 2c, the low-angle grain boundaries with misorientations ranging from 2° to 10° are marked by blue lines, while high-angle grain boundaries with misorientations ≥10° are marked by black lines. The orientation map (Figure 2d) shows a relatively random crystallographic texture, with a statistically determined average grain size of 11.3 ± 4.5 µm. Figure 2e shows the kernel average misorientation (KAM) map with a scale ranging from 0° to 5°. The green-colored regions correspond to areas with high KAM values where dislocations accumulate and are predominantly concentrated at lath boundaries and grain boundaries. Correspondingly, the geometrically necessary dislocation (GND) density map (Figure 2f) shows generally low dislocation density (5.0 × 10^14^ m^−2^) across the matrix, with locally elevated values confined to phase boundaries. It indicates that the overall dislocation density of the austempered specimen remains relatively low.

**Figure 3:**
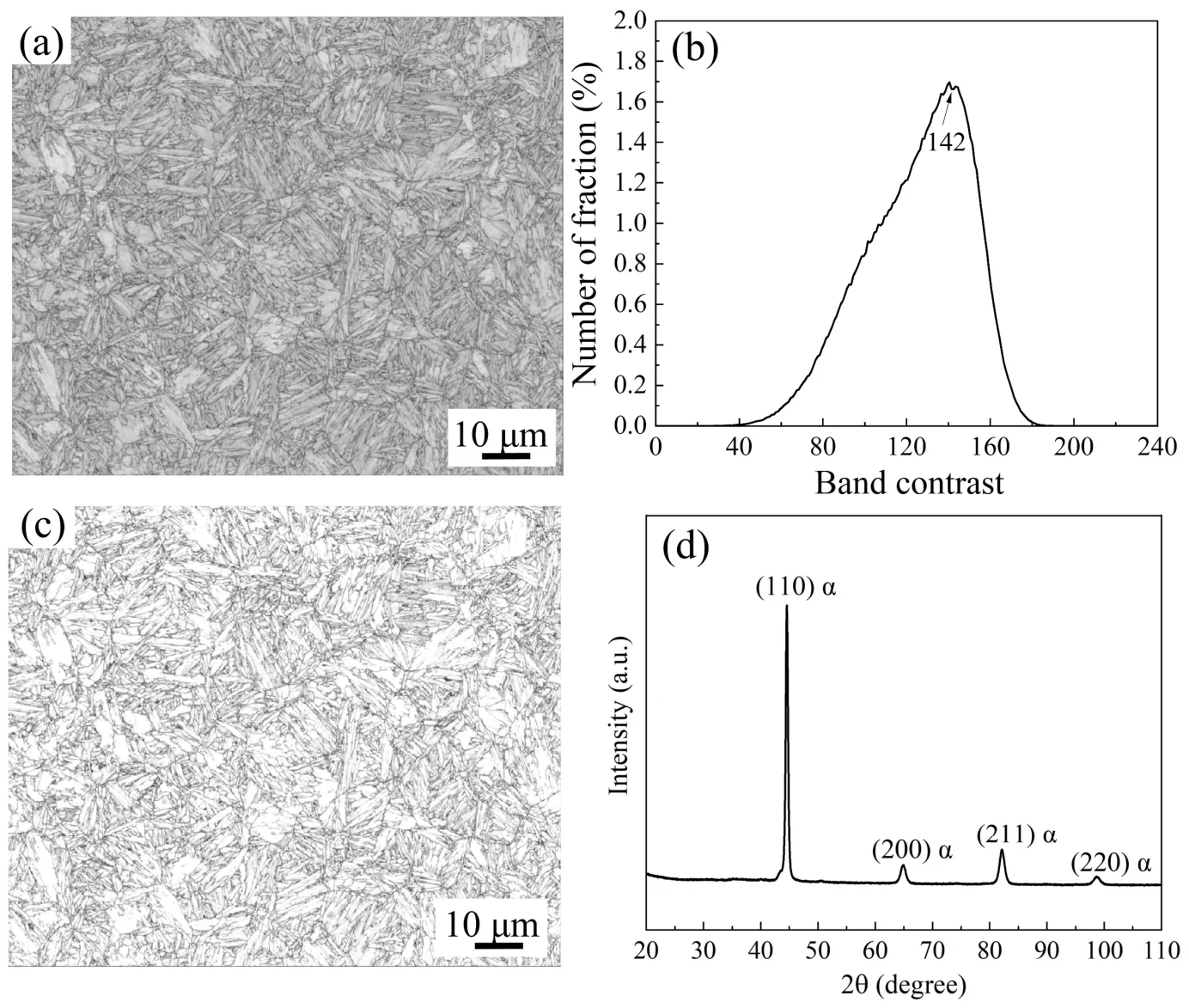
EBSD and X-ray diffraction results of the isothermally quenched specimen maps of the isothermally quenched specimen: (**a**) band contrast (BC) map; (**b**) BC curves; (**c**) phase distribution map; (**d**) X-ray diffraction pattern.

To quantify the volume fraction of martensite versus lower bainite, EBSD band contrast (BC) mapping was employed, exploiting the higher degree of lattice distortion in martensite relative to lower bainite, which is reflected in the quality of the corresponding Kikuchi diffraction patterns [30]. In the BC map (Figure 3a), regions of low BC value (dark) correspond to martensite, characterized by severe lattice distortion and high dislocation density, whereas regions of high BC value (bright) correspond to lower bainite. The threshold separating the two phases was defined as the intersection point of their respective BC value distribution curves (Figure 3b) [12]. Based on this threshold, martensite (black) and lower bainite (white) regions were segmented in Figure 3c, yielding volume fractions of 62.8% bainite and 37.2% martensite. The XRD result in Figure 3d verifies the absence of detectable retained austenite.

**Figure 4:**
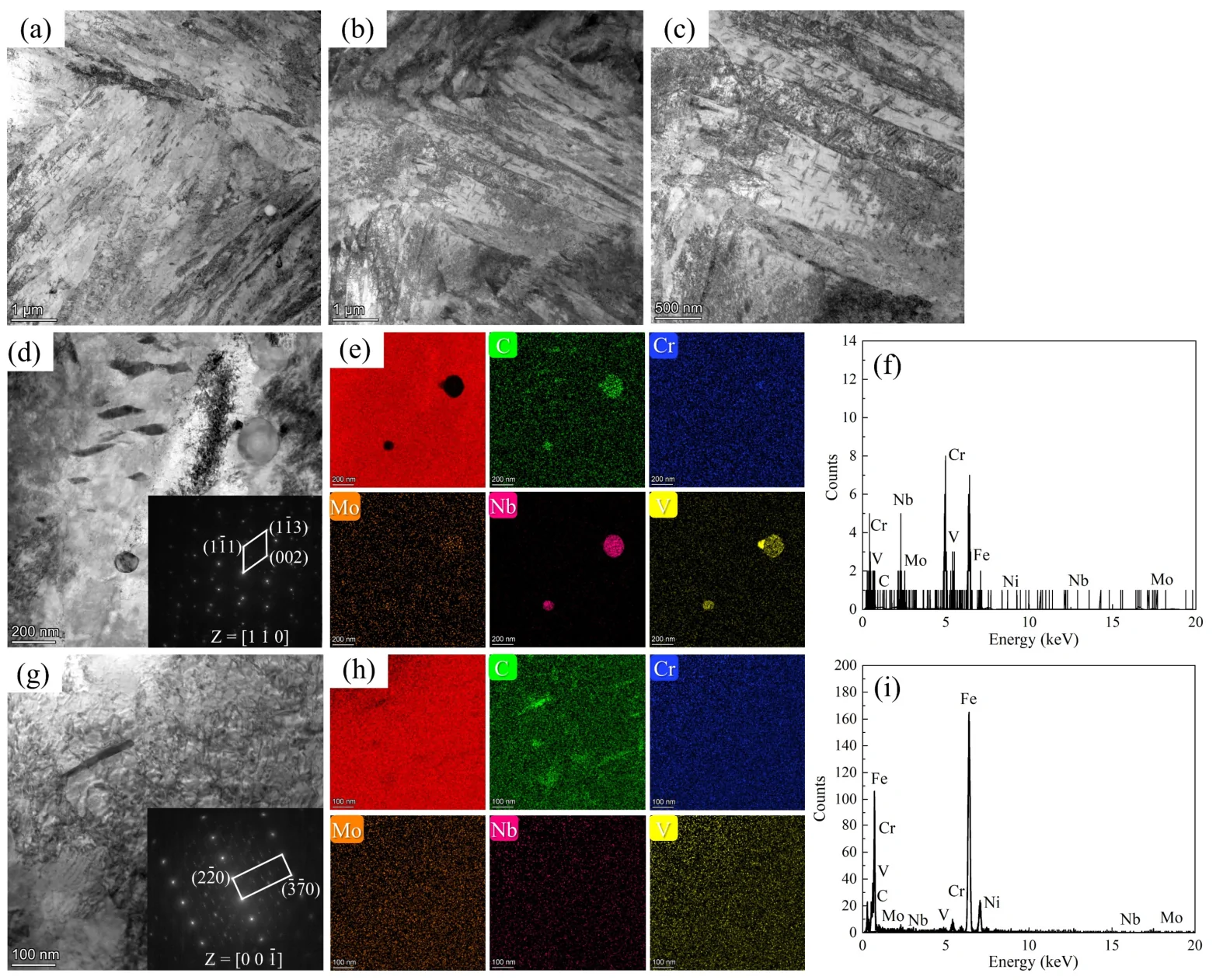
TEM micrographs of austempered experimental steel. (**a**–**c**) Matrix TEM images; (**d**) high-magnification view of spherical MC carbides; (**e**,**f**) EDS mapping and spectrum of spherical carbides in (**d**); (**g**) TEM image of rod-like M_3_C carbides; (**h**,**i**) EDS mapping and spectrum of rod-like carbides in (**g**).

Figure 4 shows the TEM characterization of the austempered microstructure. Carbides with acicular, spherical, and rod-like morphologies can be observed. The selected-area electron diffraction (SAED) patterns of MC-type and M_3_C-type carbides are presented in Figure 4d and Figure 4g, respectively. Spherical precipitates (20–80 nm) were identified by TEM-EDS mapping as MC-type carbides enriched in V and Nb (Figure 4e,f), while rod-like precipitates (~120 nm in length, Figure 4g) were identified by STEM-EDS as M_3_C-type carbides enriched in Cr (Figure 4h,i).

**Figure 5:**
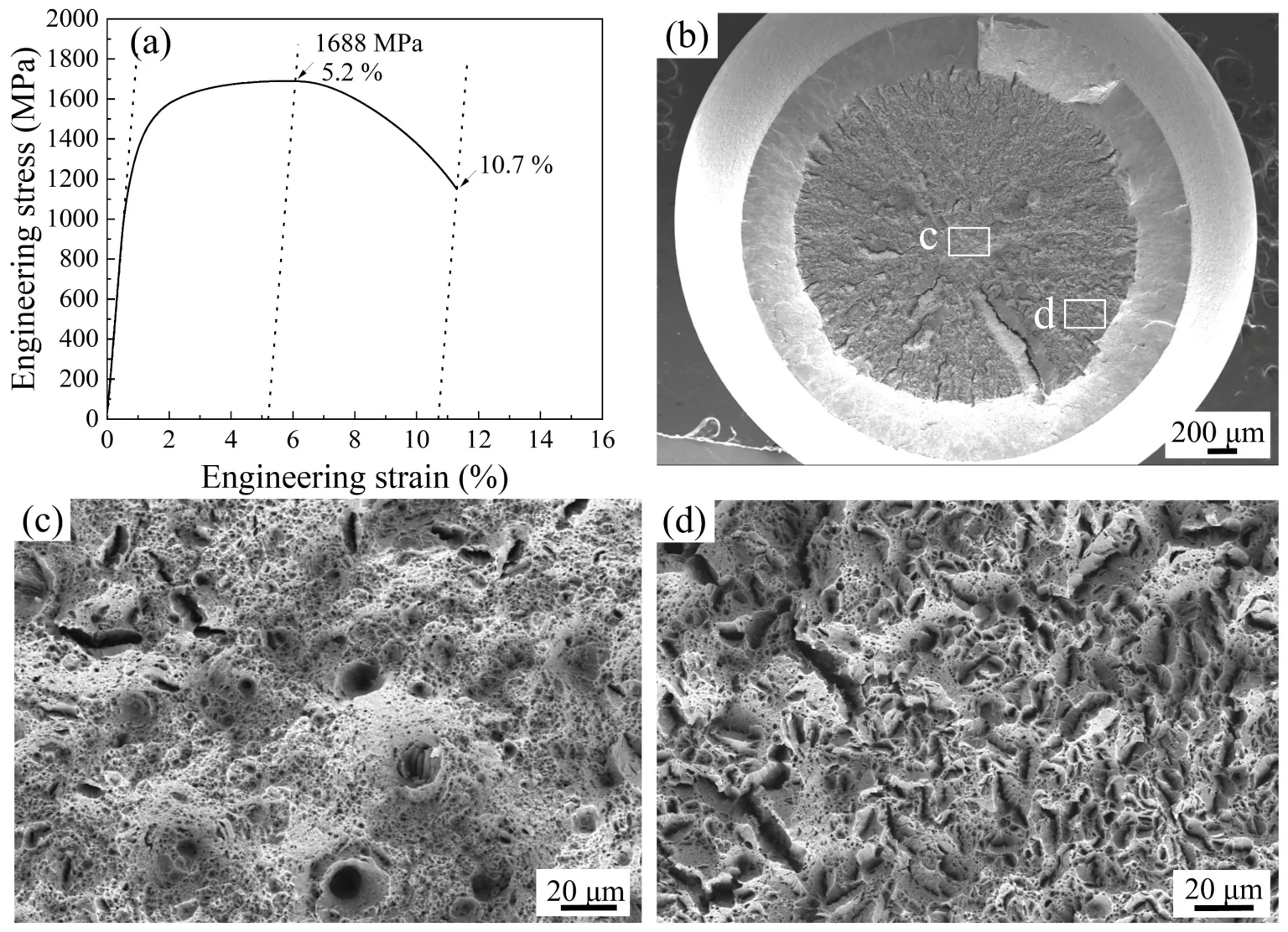
Mechanical properties and tensile fracture morphologies of the austempered experimental steel: (**a**) engineering stress–strain tensile curve; (**b**) macroscopic cup-and-cone fracture morphology of the tensile sample; (**c**) high-magnification SEM image of the central fibrous zone (marked c in (**b**)); (**d**) high-magnification SEM image of the shear lip and crack propagation zone (marked d in (**b**)).

The engineering stress–strain curve of the austempered steel is shown in Figure 5a. Following a steep elastic stage, the material exhibits extensive plastic deformation, reaching an ultimate tensile strength of 1688 MPa, with an elongation to fracture of 10.7 ± 0.6% and a reduction in area of 56.4 ± 3.5%. This good balance of strength and ductility originates from the synergistic effect of the lower bainite–martensite dual-phase microstructure: the hard martensite provides high strength, while the lower-bainite laths, with their moderate dislocation density, accommodate plastic deformation and hinder crack propagation. The Charpy V-notch impact energy at −40 °C is 10.4 ± 1.2 J. Figure 5b–d show the fracture morphologies of the investigated material. It exhibits typical ductile dimple fracture, with numerous dimples together with occasional microcracks distributed across the fracture surface. The overall fracture presents a classic cup-and-cone morphology, consisting of a central fibrous zone encircled by an outer shear-lip region that accounts for 45.2% of the total fracture area (Figure 5b). The central fibrous zone contains abundant shallow dimples and secondary cracks (Figure 5c), while the surrounding crack propagation zone exhibits distinct radial macrocracks that initiate near the center and propagate outward into the peripheral shear-lip region (Figure 5d).

### 3.2. Slow Strain Rate Tensile Testing Under Hydrogen Charging Condition

**Figure 6:**
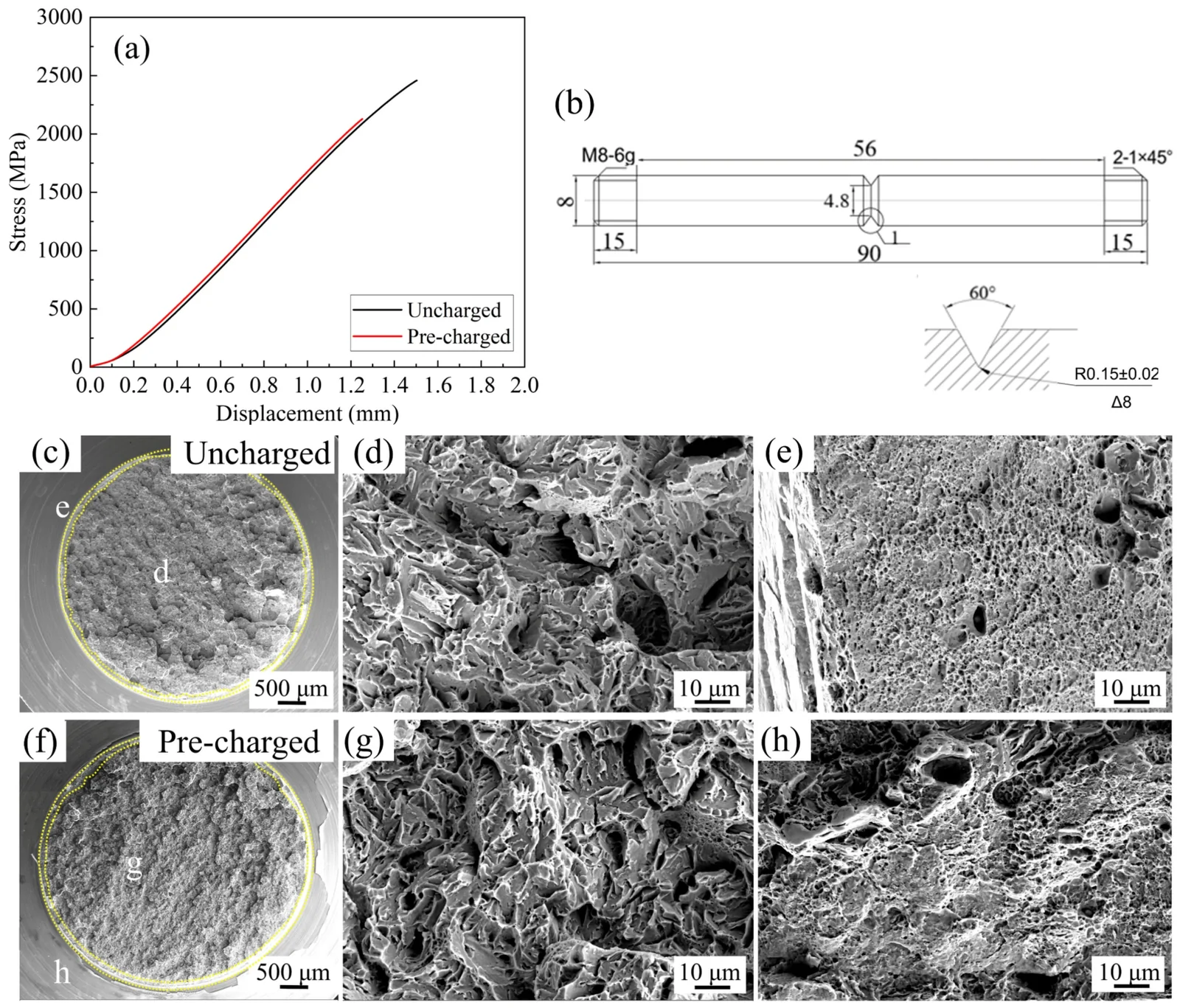
Mechanical properties and SSRT fracture morphologies of the experimental steel: (**a**) stress–displacement curves; (**b**) geometry of SSRT notched specimen; (**c**–**e**) fracture morphologies of uncharged specimens after SSRT tests; (**f**–**h**) fracture morphologies of hydrogen-precharged specimens after SSRT tests.

Figure 6a shows the stress–displacement curves obtained from SSRT testing on notched tensile specimens (Figure 6b). The uncharged specimen exhibits a notched tensile strength of 2459 ± 8 MPa, considerably higher than the ultimate tensile strength obtained from smooth specimens (Section 3.1), owing to the pronounced triaxial stress state and stress concentration developed at the notch root [3]. After hydrogen pre-charging, the notched tensile strength decreases to 2127 ± 18 MPa, yielding a HEI of 13.5%. Figure 6c–h compare the fracture morphologies of the uncharged and hydrogen-precharged specimens after the SSRT test. For the uncharged specimen, low-magnification observation reveals a shear-lip region densely populated with dimples at the notch root, consistent with typical ductile fracture (Figure 6c,e). At higher magnification, the central fracture zone displays a mixed fracture mode combining quasi-cleavage facets and dimples, together with local tearing ridges and occasional microcracks, indicating that appreciable plastic deformation accompanied crack propagation (Figure 6d). Hydrogen pre-charging alters the fracture morphology. Near the notch root, the dimple-dominated ductile region observed in the uncharged specimen is partially replaced by localized brittle fracture areas (Figure 6h). In the central fracture zone, quasi-cleavage facets become more extensive and are accompanied by numerous secondary cracks (Figure 6g). Overall, hydrogen charging reduces the notched tensile strength and shifts the fracture mode from ductility-dominated to brittleness-dominated failure, reflecting the characteristic features of hydrogen-induced delayed fracture in this steel.

### 3.3. Thermal Desorption Spectroscopy and Hydrogen Permeation Behavior Analysis

**Figure 7:**
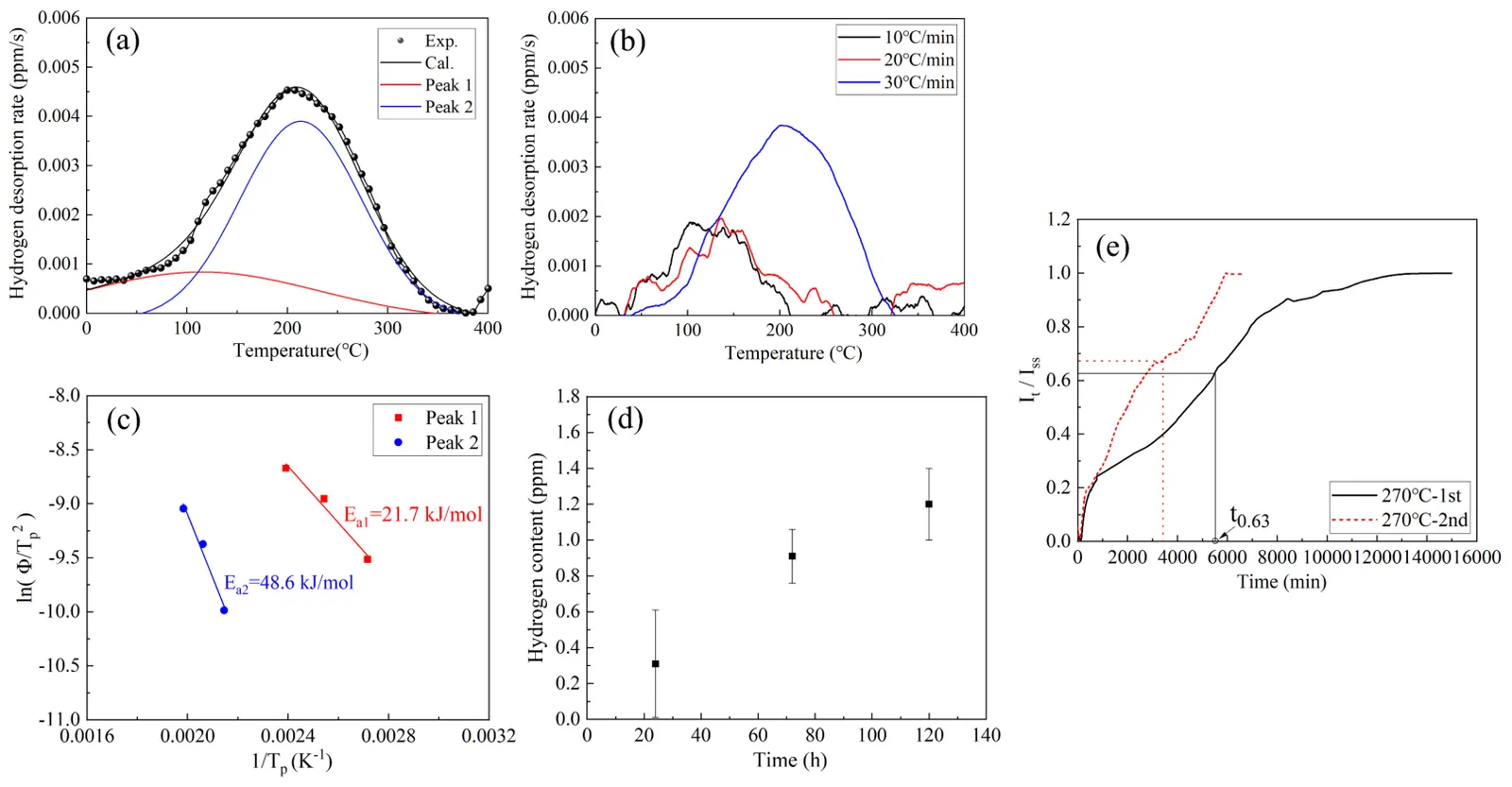
Thermal desorption spectroscopy and hydrogen permeation analysis of the investigated steel: (**a**) peak deconvolution of the TDS profile; (**b**) TDS profiles under multiple heating rates; (**c**) Kissinger plot of ln(Φ/T_p_^2^) versus 1/T_p_ for the calculation of hydrogen desorption activation energy; (**d**) hydrogen content as a function of hydrogen charging periods; (**e**) cyclic hydrogen permeation transient curves.

Figure 7a presents the TDS profile of the specimen austempered below the *M_s_* temperature, measured at a heating rate of 30 °C/min. A thermal desorption analysis (TDA) peak can be regarded as the superposition of multiple sub-peaks, each corresponding to hydrogen release from trapping sites with different hydrogen-binding energies [31]. Variations in the overall desorption profile and peak temperature result from the relative contributions of different types of trapping sites and their amount, which consequently influence HE behavior. It is necessary to apply peak deconvolution to distinguish the contributions of different trapping sites. The TDA profile can be decomposed according to the following equation [32]:

(7) $$F\left(x\right)=\sum a_{i}\cdot exp({-\left(x-i\right)}^{2}/b_{i}^{2})$$

where F(x) represents the hydrogen desorption rate (ppm/s), x is temperature (°C), i denotes the position of the sub-peak, and a_i_ and b_i_ are the parameters related to the peak height and width, respectively. The experimental desorption profiles were deconvoluted by Gaussian fitting into two sub-peaks (Peak 1 and Peak 2), corresponding to hydrogen releasing from different trapping sites. As shown in Figure 7b, both deconvoluted sub-peaks exhibit a noticeable shift toward higher temperatures with increasing heating rate. This shift in peak temperature is a typical kinetic feature of thermally activated hydrogen desorption. Based on the relationship between peak temperature and heating rate, the desorption activation energy associated with each type of hydrogen-trapping site was quantitatively determined according to the Kissinger-type plots of ln(φ/Tp^2^) versus 1/Tp shown in Figure 7c. The activation energies *E*a of Peak 1 and Peak 2 were determined to be 21.7 kJ/mol and 48.6 kJ/mol, respectively, corresponding to hydrogen-trapping sites with weak binding energy (such as interstitial sites, dislocations and grain boundaries) and relatively high binding energy (such as semi-coherent interfaces) [33]. Figure 7d further reveals that hydrogen content rises continuously with prolonged charging duration.

Electrochemical hydrogen permeation tests were performed to further characterize the hydrogen diffusion behavior of the steel. The normalized hydrogen permeation curves are shown in Figure 7e. The specimen thickness was 0.975 mm and the time to reach t_0.63_ was 330,145 s in the first hydrogen permeation cycle. In the second cycle, the specimen thickness was 0.933 mm, and the time to reach t_0.63_ declined to 203,450 s. Hydrogen permeation current density gradually increased and finally plateaued into a steady-state condition with prolonged charging period. During the first permeation cycle, hydrogen atoms simultaneously occupy both reversible and irreversible trapping sites within the matrix. Because irreversible traps exhibit strong hydrogen-binding capacity, a substantial fraction of the charged hydrogen becomes immobilized and does not readily desorb, which considerably delays the attainment of steady-state permeation. The apparent hydrogen diffusivity calculated from the first-cycle transient using Equation (3) is 4.79 × 10^−9^ cm^2^/s. In the second permeation cycle, however, the irreversible traps, which were saturated during the first cycle, cannot capture hydrogen further. Only reversible traps remain active. The 24 h rest period between cycles allows hydrogen retained in reversible traps from the first cycle to fully desorb, thereby providing consistent initial conditions for the second measurement.

From the second-cycle permeation data, the apparent hydrogen diffusivity was calculated to be 7.13 × 10^−9^ cm^2^/s using Equation (3), which is markedly higher than that obtained from the first cycle. This increase is consistent with the irreversible hydrogen-trapping sites having been largely occupied during the first permeation cycle. The corresponding total trap density (*N*_T_) and irreversible trap density (*N*_ir_) were determined to be 2.86 × 10^22^ cm^−3^ and 0.94 × 10^22^ cm^−3^, respectively. These results indicate that the carbides precipitated during austempering act as efficient irreversible hydrogen-trapping sites, effectively capturing and immobilizing hydrogen and thereby suppressing its diffusion and local accumulation within the steel matrix.

## 4. Discussion

### 4.1. Effect of Austempered Microstructure on Hydrogen Embrittlement Susceptibility

The investigated materials exhibit a martensite/bainite microstructure with dispersedly precipitated MC-type and M_3_C-type carbides (Figure 2, Figure 3 and Figure 4). Fresh martensite formed at the austempering temperature undergoes low-temperature tempering, whereby severe lattice distortion and high dislocation density are partially alleviated [21]. The bainitic phase transformed from retained austenite during austempering induces substantial stress relaxation and preserves plasticity [12]. Moreover, the phase interfaces further modify the hydrogen diffusion pathways, mitigating local hydrogen enrichment and thereby improving resistance to HE at the microstructural scale [13,34]. The HEI of the austempered specimen reaches 13.5%, while the HEI from the same material under quenched and tempered conditions (tempered at 500 °C, 580 °C and 620 °C for 2h respectively) ranges from 46.7% to 62.8% [35], demonstrating that the austempered microstructure has obviously better HE resistance compared to the martensitic microstructure from quenched and tempered conditions at a similar strength level. Fang et al. [3] also reported that a quenched and tempered steel at the strength level of 1200 MPa obtained a HEI of 30.9% with 1.42 ppm precharged hydrogen, further highlighting the better HE resistance of the austempered martensite/bainite dual-phase microstructure. This observation is consistent with the relatively low apparent hydrogen diffusion coefficient (4.79 × 10^−9^ cm^2^·s^−1^) measured via hydrogen permeation tests on the material.

In addition to the phase interfaces, the finely dispersed MC-type and M_3_C-type carbides in the matrix can act as effective hydrogen-trapping sites that regulate hydrogen diffusion and enrichment [33]. First-principles calculations by Kawakami et al. [36] demonstrated that M_3_C/ferrite interfaces act as reversible hydrogen-trapping sites; abundant, finely dispersed cementite can continuously trap free hydrogen and reduce the overall hydrogen diffusion rate. Nano-sized V-rich MC carbides, in contrast, function as strong (essentially irreversible) hydrogen traps, and together these two types of carbides form a multistage hydrogen-trapping system [37]. This multistage hydrogen-trapping behavior is further supported by the TDS results shown in Figure 7a–c. The desorption spectrum can be well resolved into two sub-peaks. Peak 1 (*E*a = 21.7 kJ/mol) is associated with weak reversible traps, such as interstitial sites, dislocations and grain boundaries, whereas Peak 2 (*E*a = 48.6 kJ/mol) could be high-energy reversible traps associated with M_3_C/ferrite interfaces.

Fang et al. [3] found in a V/Nb microalloyed medium-carbon high-strength bolt steel that the synergistic combination of homogeneously precipitated M_3_C cementite and nano-sized V-rich MC carbides markedly lowers the effective hydrogen diffusion coefficient, suppresses hydrogen accumulation at prior-austenite grain boundaries and notch tips, and mitigates hydrogen-induced intergranular brittle fracture. Consistent with this trapping behavior, the hydrogen content in the present steel increases continuously with charging duration up to 120 h (Figure 7d), while the normalized permeation curves (Figure 7e) exhibit a pronounced delay before reaching the steady-state current, reflecting the strong hydrogen-trapping capacity of the dispersed carbides and their effectiveness in postponing hydrogen saturation at defect sites.

### 4.2. Hydrogen-Induced Delayed Fracture Mechanism

The SSRT fracture surface (Figure 6f) exhibits pronounced spatial inhomogeneity, suggesting that both the hydrogen distribution and the stress field at the notch tip are markedly non-uniform, with hydrogen inhomogeneity being the dominant factor governing premature failure. The maximum penetration depth of hydrogen within a specimen of finite thickness over a charging time t can be estimated from the semi-infinite diffusion equation [38]:

(8) $$x_{max}=\sqrt{2Dt}$$

where D is the hydrogen diffusion coefficient (cm^2^·s^−1^), and t is the charging time (s). For hydrogen to diffuse from the specimen surface to the root of the V-notch over a minimum distance of 2.4 mm within the 24 h pre-charging period, the required minimum hydrogen diffusion coefficient is 3.33 × 10^−7^ cm^2^·s^−1^. This threshold is roughly two orders of magnitude higher than the apparent hydrogen diffusion coefficient measured in Section 3.3, indicating that hydrogen introduced during pre-charging does not fully penetrate to the specimen core within 24 h. Consequently, under subsequent SSRT loading, hydrogen progressively accumulates in the high-stress notch region, where the local hydrogen concentration becomes the key factor controlling the HE behavior [39].

The steep stress gradient generated near the notch root during SSRT drives further hydrogen diffusion toward this high-stress region [40]. Once the local hydrogen concentration exceeds a critical threshold, hydrogen-induced cracks nucleate and propagate under the combined action of stress and hydrogen, ultimately triggering delayed fracture. The low hydrogen diffusion coefficient, by restricting hydrogen redistribution, favors localized hydrogen accumulation and is consistent with the quasi-cleavage fracture features observed in the specimen center (Figure 6g).

**Figure 8:**
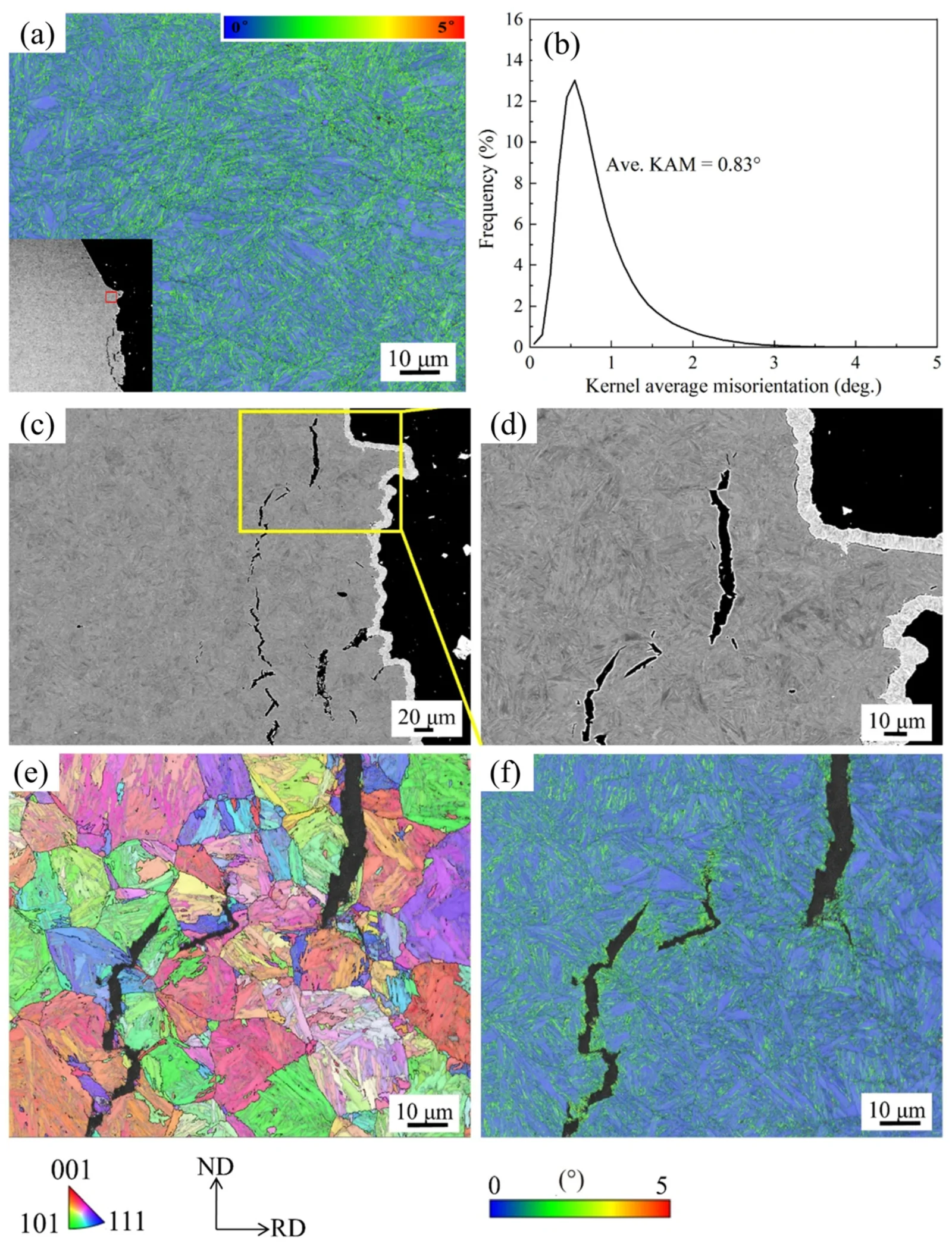
ECCI-EBSD correlative characterization of the fracture cross-section in the hydrogen-precharged SSRT specimen: (**a**) KAM map; (**b**) corresponding KAM distribution curve; (**c**) ECCI image near the notch root; (**d**) magnified ECCI image of the region marked in (**c**); (**e**,**f**) corresponding IPF and KAM maps of the region marked in (**c**), respectively.

Figure 8a,b present the KAM map and corresponding statistics at the notch root of the SSRT specimen. Since the KAM value reflects the local deformation strain, the elevated KAM values observed at the notch root (average 0.83°, Figure 8b) strongly demonstrate that severe plastic strain localization, intensive dislocation accumulation, and obvious stress concentration preferentially occur at the notch root under external tensile loading. Correlative ECCI/EBSD characterization of the cross-sectional fracture region of the hydrogen-precharged specimens (Figure 8c–f) provides direct microstructural evidence for the hydrogen-induced crack initiation and propagation behavior. Numerous microcracks preferentially nucleate and accumulate near the high-strain notch root region (Figure 8c,d), and these microcracks mainly propagate in a transgranular mode through the martensite/bainite dual-phase microstructure (Figure 8e). This behavior is consistent with the strain-localization characteristics during tensile deformation, in which heterogeneous plastic deformation is concentrated in localized high-strain regions rather than being uniformly distributed throughout the microstructure. Such localized deformation can promote preferential crack initiation and facilitate crack initiation and subsequent propagation in these highly strained, defect-rich regions [41]. Furthermore, the hydrogen-assisted fracture process may involve the coupled contributions of hydrogen-enhanced localized plasticity (HELP) and hydrogen-enhanced decohesion (HEDE) mechanisms, which accelerate strain localization and crack propagation process. Hydrogen dissolved in the lattice can further intensify local strain localization by promoting dislocation multiplication and motion [42]. Specifically, diffusible hydrogen atoms aggregate around movable dislocations to form stable Cottrell atmospheres, which significantly reduces the dislocation pinning effect and facilitates dislocation emission, glide and accumulation. The accumulation of massive dislocations at microcrack tips further increases the local internal stress field and lattice distortion. Meanwhile, hydrogen segregation at grain boundaries and phase interfaces could reduce the atomic bonding force through the HEDE mechanism. The coupling of the two mechanisms jointly promotes the continuous initiation and propagation of microcracks, and eventually induces macroscopic fracture.

The numerous martensite–bainite lath boundaries can trap diffusive hydrogen and alter its diffusion pathways. Meanwhile, nanoscale MC and M_3_C carbides dispersed within both phases provide effective hydrogen-trapping sites, further restricting hydrogen transport toward dislocations and grain boundaries. Consequently, the investigated steel exhibits a relatively low hydrogen diffusivity of 4.79 × 10^−9^ cm^2^/s. Comparing to a conventional fully martensitic microstructure [3,35], the stress-relieved bainitic and tempered martensitic microstructure exhibits moderate dislocation density and can partially alleviate local stress concentrations during deformation. These microstructure features may therefore partially mitigate HEDE-induced interfacial decohesion.

## 5. Conclusions

This study investigated the microstructural, mechanical properties and HE susceptibility of a martensitic/bainitic dual-phase steel via austempering below the *M_s_* temperature. The main conclusions are summarized as follows:

(1) Austempering below the *M_s_* temperature induces a uniform martensitic/bainitic microstructure (37.2 vol.% martensite, 62.8 vol.% bainite) with no retained austenite, together with dispersed MC-type (V/Nb-rich) and M_3_C-type (Cr-rich) carbides. The steel achieves an ultimate tensile strength of 1688 MPa, an elongation of 10.7%, and a reduction in area of 56.4%, with mixed dimple/quasi-cleavage fracture on both tensile and impact fracture surfaces.
(2) SSRT and hydrogen permeation tests reveal the material has an HEI of 13.5%. The fracture mode shifts from ductile- to brittle-dominated. The dispersed MC-type and M_3_C-type carbides act as effective hydrogen traps that lower the effective hydrogen diffusion coefficient and immobilize diffusible hydrogen, suppressing its accumulation at high-stress sites such as notch tips and grain boundaries, thereby reducing the overall HE susceptibility.
(3) HE is generally considered to involve the coupled contributions of the HELP and HEDE mechanisms, in which stress-assisted hydrogen accumulation at highly stressed regions can promote crack initiation and propagation. The martensitic/bainitic phase boundaries can modify hydrogen diffusion pathways and redistribute local stresses, while carbides provide additional hydrogen-trapping sites. These microstructural features may reduce local hydrogen accumulation and strain localization, thereby contributing to the improved HE resistance of this ultra-high-strength steel.

## Figures and Tables

**Table 1:** Chemical composition of the investigated 31CrMoNiNbV steel (wt.%).

C	Si	Mn	P	S	Cr	Ni	Mo	Nb	V	Fe
0.31	0.04	0.19	0.008	0.001	3.01	0.92	2.00	0.085	0.460	Bal.

## Data Availability

The original contributions presented in this study are included in the article. Further inquiries can be directed to the corresponding author.
